# Environmental contamination with feces of free-roaming dogs and the risk of transmission of *Echinococcus* and *Taenia* species in urban regions of southeastern Iran

**DOI:** 10.1186/s13071-024-06435-x

**Published:** 2024-08-23

**Authors:** Saeedeh Shamsaddini, Carina Schneider, Sonja Dumendiak, Hossein Aghassi, Hossein Kamyabi, Elham Akhlaghi, Marion Wassermann, Majid Fasihi Harandi, Peter Deplazes, Thomas Romig

**Affiliations:** 1 Research Center for Hydatid Disease in Iran, Afzalipour School of Medicine, Kerman University of Medical Sciences, Kerman, 76169114115 Iran; 2 Dept of Parasitology, Hohenheim University, 70599 Stuttgart, Germany; 3 Dept of Medical Parasitology, Afzalipour School of Medicine, Kerman University of Medical Sciences, Kerman, 7616914115 Iran; 4 Vetsuisse and Medical Faculty, Institute of Parasitology, University of Zurich, Zurich, Switzerland; 5 Dept of Gastroenterology and Hepatology, University Hospital Zurich, Zurich, Switzerland

**Keywords:** *Echinococcus*, Cystic echinococcosis, Taeniidae, Urbanization, Free-roaming dog, Environmental contamination

## Abstract

**Background:**

Dogs are the most important definitive hosts of zoonotic taeniid helminths worldwide. Different *Echinococcus* and *Taenia* species of domestic and wild carnivores pose a potential risk to human population. High populations of free-roaming dogs (FRDs) in urban areas of Iran and widespread contamination of the environment with dog feces is a potential source of infecting people living in the urban regions with cystic echinococcosis (CE). Our knowledge on the risk of CE transmission in the urban settings in the endemic regions is limited. The present study surveyed the species and genotypes of *E. granulosus* sensu lato and other taeniids by examining feces of free-roaming dogs in the urban areas in the city of Kerman, southeastern Iran.

**Methods:**

The city was divided into 100 consecutive blocks of which 25 blocks were randomly selected. Fecal samples of FRDs were counted, mapped and fresh samples were collected. Then Zinc chloride flotation, and sequential sieving was performed, and the samples were examined under an inverted microscope. Single individual taeniid eggs were isolated, partial *nad1* gene was amplified and sequenced to identify species and genotypes.

**Results:**

In total 5607 fecal samples of dogs were mapped and 83 fresh samples were collected. Taeniid eggs were detected in nine fecal samples (10.8%) from seven out of the 25 city blocks (28.0%). *Echinococcus* eggs were found in four samples (4.8%) from three city blocks, two samples containing *E. granulosus* sensu stricto (2.4%), two samples containing *E. canadensis* G6/7 (2.4%). In addition, three samples contained eggs of *Taenia hydatigena* (3.6%), and one sample of *Taenia serialis* (1.2%).

**Conclusions:**

This study documented the potential risk of CE transmission to humans resulting from the feces of dogs roaming freely in urban areas.

**Graphical Abstract:**

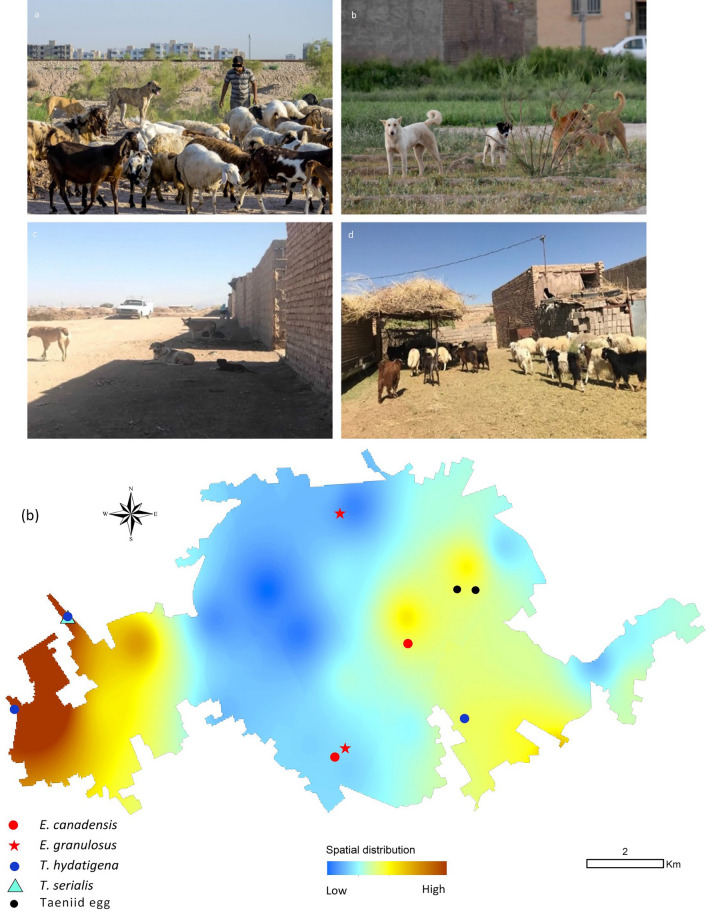

**Supplementary Information:**

The online version contains supplementary material available at 10.1186/s13071-024-06435-x.

## Background

Different taeniid tapeworms are significant zoonotic parasites of humans and animals. Taeniids are mainly transmitted via predator–prey interactions between domestic and wild carnivorous animals as definitive hosts and herbivores and/or omnivores as intermediate hosts [1, 2]. Dogs are the most important definitive domestic hosts of zoonotic taeniid cestodes all over the world [3]. Several species of *Echinococcus* and *Taenia* are the agents of human and livestock infections with dogs playing an essential role as definitive hosts [3, 4].

Cystic echinococcosis (CE) is a chronic, neglected zoonotic infection caused by various species and genotypes of the *Echinococcus granulosus* sensu lato (s.l.) complex. CE is endemic in more than 100 countries worldwide [5]. In a global multi-criteria ranking of food-borne parasites, *E. granulosus* s.l. is ranked second in the list of 24 food-borne parasites [6]. The economic losses caused by echinococcosis are estimated to reach US$ 3 billion, equivalent to 0.03% of the Gross domestic product (GDP) in the endemic countries. A study in Iran showed that CE imposes an annual financial burden of more than US$ 230 million on the country’s economy [7, 8].

In Iran, human echinococcosis has been investigated in two ultrasound surveys, in the nomads of the southern province of Fars and in the rural areas of Kerman province in southeastern Iran, in which the ultrasound prevalence of echinococcosis was reported as 1.8% and 0.2%, respectively [9, 10]. The surgical incidence of CE in Iran was estimated at 1.27 per 100,000 people in the population between 2000 and 2009 and the number of asymptomatic people with CE infection in the country was estimated at 635,232 [8]. According to the Iranian Hydatid Disease Registry, a similar incidence of 1.2 per 100,000 people can be observed in Kerman province [11]. In Iran, *E. granulosus* s.l. is transmitted mainly among dogs and livestock, although there is a wide range of different intermediate host species. According to available data, the prevalence of CE in livestock ranges from 1.3% to 74.4% in sheep, 0.4% to 37.8% in goats, 1.3% to 40.1% in cattle, and 8.8% to 40% in camels [12].

As in most CE-endemic regions, the role of dogs in CE transmission is also very important in Iran. According to the available data, dogs are the most important definitive host, both in terms of population size and frequency. For example, in Kerman city, the dog: human ratio has been determined as 1.2 dogs per 100 people, with a density of 5.8 dogs per km street survey [13]. In Iran dog ownership is not encouraged and most of the dogs roaming freely in the urban regions are unowned, however field observations indicate that there are few owned dogs, roaming freely in public parks and streets [13]. The prevalence of *Echinococcus* in free-roaming dogs (FRD) in Iran varies from 7 to more than 60% depending on different geographical regions [14, 15]. In Kerman, prevalence estimates range from 6.8 to 10.7% [16, 17]. More data are required on the species and genotypes of *E. granulosus* s.l. infecting FRDs in urban areas of many endemic regions of the world. In northern Iran, the prevalence in dogs has been reported from 21.1 to 46.7% and 25% in Mazandaran and Gilan provinces, respectively [14, 15]. In a recent study on canine echinococcosis in rural regions of northwestern Iran using necropsy, Zarei et al. showed that 4% of FRDs were found infected with *E. granulosus* sensu stricto (s.s.)*,* G1 genotype [18].

Most of our knowledge on the molecular epidemiology of echinococcosis in dogs are limited to rural areas. CE is predominately thought to be a rural disease; however, relatively little is known about the nature and extent of *E. granulosus* s.l. transmission in urban settings [19]. Recent studies indicate the circulation of *E. granulosus* s.l. in cities. In the urban and suburban populations, dogs with free access to public areas are a risk to public health. In a study on dog feces in two cities in north-central Chile, Acosta Jamet et al. showed that 11.7% of samples were positive by using copro-Ag ELISA, while 3.5% were copro-positive in rural regions [20]. In the streets and green spaces of Tartu, Estonia, 2.2% of 181 samples from dogs were found infected with *E. granulosus* s.s. [21].

Studies have shown that *E. granulosus* sensu lato is a complex of cryptic species and genotypes, and this diversity may influence the epidemiology and transmission of CE [22]. Due to the great significance of dogs in the transmission of echinococcosis, molecular epidemiological studies on the infection in the definitive hosts are essential in each endemic area for a successful control program [23]. However, most genotyping studies have been performed on the parasites isolated from livestock rather than dogs in each area and due to the substantial livestock transport and trade, understanding the genotypes occurring on sub-regional scales is challenging [24, 25]. Therefore, determining the *E. granulosus* s.l. taxa present in the canine final host, is of great importance for taking necessary actions against CE in endemic areas.

More information is required on the molecular epidemiology of canine echinococcosis in urban areas of endemic countries. The purpose of the present study was to investigate the frequency, species and genotypes of *E. granulosus* s.l. and other taeniids present in the feces of free-roaming dogs in the southeastern city of Kerman, including built-up areas as well as public parks, green spaces, and cultivated lands. We have also mapped the distribution of dog feces within the city to highlight possible variance of environmental risk for the people living in the urban areas.

## Methods

### Study areas

The city of Kerman (30.29 N, 57.06 E), the capital of Kerman province, is located in the southeast of Iran with a population 548,000 and 220 km^2^ surface area (http://amar.sci.org.ir). The climate is arid and semi-arid with a mean annual precipitation of 132.4 mm. The population of free-roaming dogs has been estimated at 6781 dogs in the city [13].

**Fig. 1:**
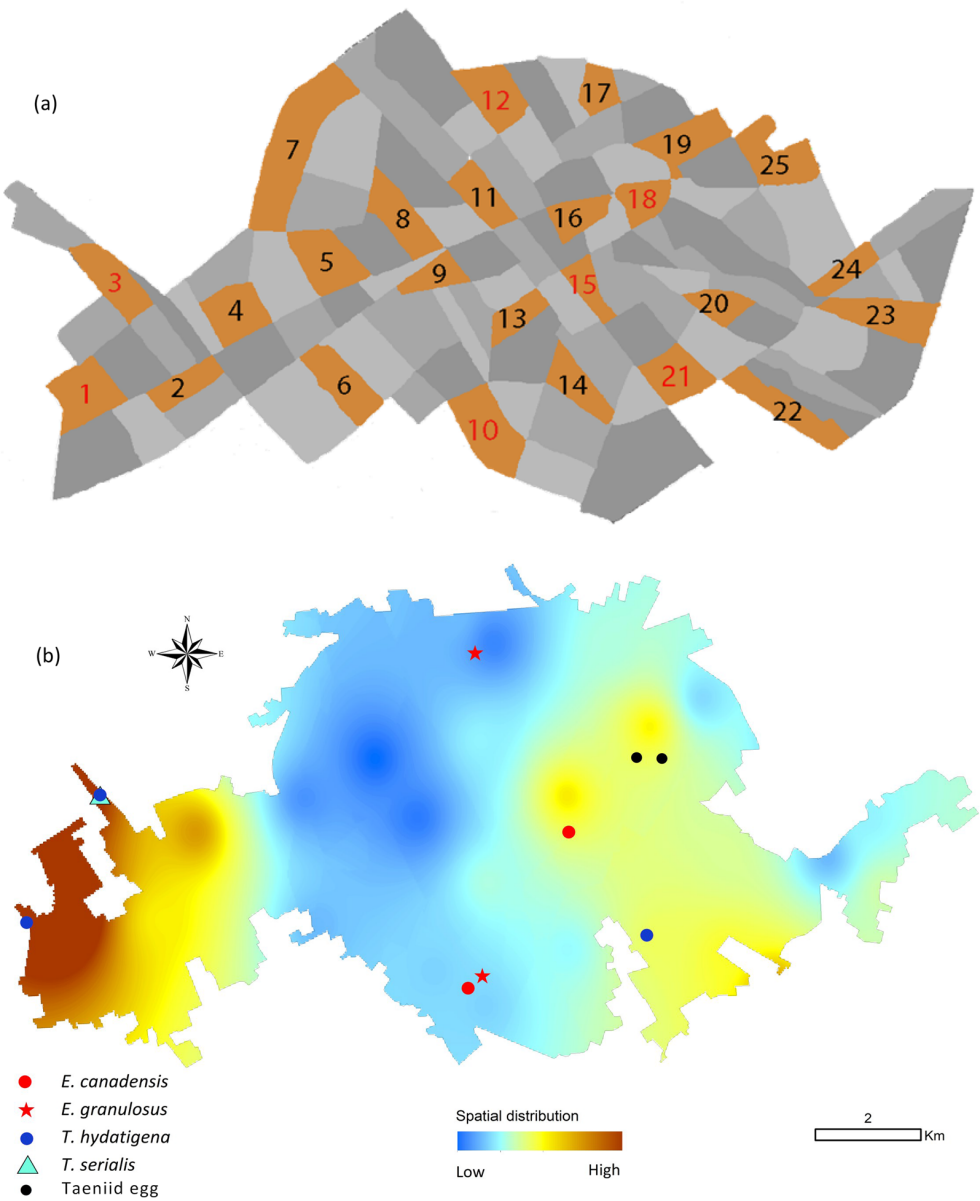
**a.** Schematic representation of the city of Kerman showing the city divided into 100 blocks. All orange blocks indicate the areas from where free-roaming dog fecal samples were collected. Taeniid eggs were found in the blocks identified by the red numbers. **b.** The density and spatial distribution of free-roaming dog feces in Kerman city and the points from where *E. granulosus* sensu stricto*, E. canadensis* G6/7*, Taenia hydatigena* and *T. serialis* eggs were detected

In accordance with the guidelines of World Animal Protection (formerly World Society for the Protection of Animals, WSPA) the city was proportionately divided into 100 consecutive blocks using municipal map with 1/100,000 scale [26]. Each block was marked with one of the four colors, green, blue, red, and orange, starting from the center of the city. Finally, by a randomly selected color, 25 urban blocks were selected (Fig. 1a) [13, 26].

### GIS data analysis

From November 2020 to December 2020 in each of the 25 selected blocks, the entire area was searched for dog feces. The dog feces were counted and the characteristics of each sample was recorded including the GPS coordinates, consistency and freshness. The density and spatial distribution of feces were plotted on the city map using ArcGIS 10.8 software using the point density. Extrapolations were made for the dog feces found in the 25 city blocks and the density of the dog feces in the 25 city blocks was extrapolated to a city-wide estimate. Kruskal–Wallis test was used to compare the frequency of dog feces in different city blocks in five regions of the city in north (block No. 12,17,18,19), east (block No. 20,22,23,24,25), south (block No. 6, 10, 14, 21), west (block No. 1, 2, 3, 4, 7), and center (block No. 5, 8, 9, 11, 13,15, 16). Significance values have been adjusted by the Bonferroni correction for multiple tests.

### Sample preparation

Dog feces were visually identified by the veterinary experts according to their shape, size, and other field signs [27, 28]. Fresh fecal samples which by appearance had been shed within the past 24 h were collected in disposable plastic containers with an ID number. To reduce the possibility of duplicate samples, each sample was taken at a minimum distance of about 200 m from other samples [16]. The samples were transferred to the lab and for safety reasons the samples were stored at -80 °C for at least two weeks [29]. In the next step, 70% ethanol at a 2∶1 volumetric ratio was added to the samples and they were passed through a double-layer sterile gauze and centrifuged for 5 min at 1600 g. The sediments were then preserved in 70% ethanol until use.

Taeniid eggs were retrieved from fecal material using zinc chloride flotation with sequential sieving [30]. In brief, the samples were centrifuged at 1600 g for 5 min and the ethanol supernatant was discarded. To remove the remaining ethanol, distilled water was added to the sediment and the resulting suspension was centrifuged again at 1600 g for 5 min. The supernatant was discarded and zinc chloride solution with a specific gravity of 1.45 g/cm^3^ was added to the sediment in a ratio 1:5 (v/v). The resulting suspension was centrifuged at 400 g for 30 min. The supernatant solution was successively passed through sieves with mesh size of 50 μm and 20 μm, respectively. Taeniid eggs pass through the 50 µm sieve and are retained by the 20 µm sieve [31]. The 20 µm sieve with the eggs was inverted and washed thoroughly with water. The liquid containing the eggs was collected in a 50 ml tube. The 50 ml tubes were centrifuged again at 1600 g for 10 min. The supernatant was carefully removed until 1–2 ml remained, the pellet with eggs was dissolved and transferred to 2 ml tubes and stored at 4 °C until further processing.

### DNA amplification and sequencing

To detect taeniid eggs, the suspension was transferred to a petri dish and placed under an inverted microscope. The complete sample was carefully screened. In case of positive samples, taeniid eggs were isolated individually using a micropipette with a volume of 1 µl, transferred into 9 μl 0.02 M NaOH solution, and lysed at 95 °C for 10 min [31]. The lysate was used as template for PCR amplification. To identify the species of taeniid egg, partial NADH dehydrogenase subunit 1 (*nad1*) gene was amplified by nested PCR, using the following primer pairs as outside and inside primers: forward-out 5′ -TGTTTTTGAGATCAGTTCGGTGTG-3′, reverse-out 5′ -CATAATCAAACGGAGTACGATTAG-3′, and forward-in 5′-CAGTTCGGTGTG CTTTTGGGTCTG-3′ and reverse-in 5′-GAGTACGATTAGTCTCACACAGCA-3′ [31]. The amplicons were visualized by electrophoresis on 1.5% agarose gel stained with GelRed™ (Hayward, USA). Nested PCR products were purified according to the instructions of the High Pure PCR Product Purification Kit (Roche, Germany) and resulting fragments were subjected to Sanger sequencing (Microsynth Seqlab GmbH, Göttingen, Germany) in reverse directions. Sequences were viewed and edited using GENTle V1.9.4 software (Manske M., University of Cologne, Germany) and compared with GenBank entries using NCBI BLAST online program to identify the species/genotype.

## Results

**Fig. 2:**
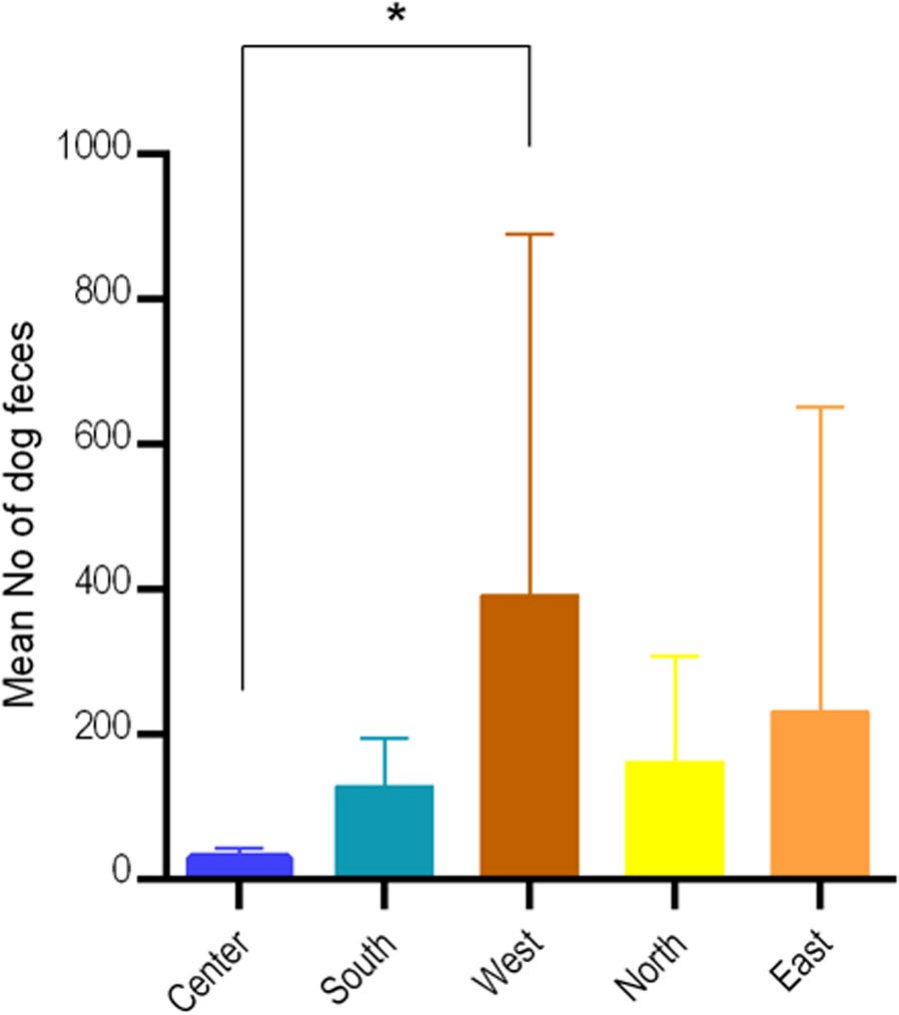
Frequency distribution of mean number of dog fecal samples observed in different city blocks in five regions of the city i.e. north, east, south, west, and center. Kruskal–Wallis test (*H* = 8.96, *df* = 4, *p = 0.04)

Within the 25 selected blocks in the city, 5607 dog feces were counted in total, and 83 fresh samples were collected. Table 1 shows the frequency distribution of the dog feces and the samples collected in the city of Kerman. In total, nine (10.8%) of the fecal samples from seven out of 25 city blocks (28.0%) contained taeniid eggs. Four species of taeniids were identified using PCR-sequencing of the *nad1* gene, namely *Echinococcus granulosus* sensu stricto (2.4%), *E. canadensis* G6/7 (2.4%), *Taenia hydatigena* (3.6%) and *T. serialis* (1.2%). The sequence data of the taeniids found in the study is provided in Table 2. In one block in the southern parts of the city, *E. canadensis* G6/7 and *E. granulosus* s.s. occurred together. Also, one dog sample showed a mixed infection with *T. hydatigena* and *T. serialis*. Figure 1b shows density and spatial distribution of dog feces and geographical locations of the egg-positive samples in the city of Kerman. Analysis of the density of dog feces showed there are 101.9 dog feces per km^2^ of the city surface area. The density distribution of 5607 FRD feces presented several major hotspots in the city (Fig. 1b), demonstrating potentially high-risk areas of the city for CE transmission. Kruskal–Wallis test demonstrated a significant difference (*H* = 8.96, *df* = 4, *p* = 0.04) between the frequency distribution of dog feces in the blocks of the center and west of the city (Fig. 2).

**Table 1:** Frequency distribution of taeniid eggs found in the microscopic and molecular study of the fecal samples collected from free-roaming dogs in 25 randomly selected blocks in the city of Kerman, southeastern Iran

Species (*n* eggs)	*n* samples (*n* eggs) positive by molecular method	*n* taeniid-egg positive samples (*n* eggs isolated)	*n*^a^ feces detected (*n* fresh feces collected)	Block ID
*T. hydatigena* (10)	1 (10)	1 (19)	952 (10)	1
			238 (5)	2
*T. hydatigena* (1)*, T. serialis* (2)	1 (3)	1 (7)	397 (4)	3
–	0	0	17 (0)	4
–	–	–	43 (0)	5
–	–	–	200 (1)	6
–	–	–	845 (7)	7
–	–	–	11 (0)	8
–	–	–	7 (0)	9
*E. canadensis* G6/7 (3)*, E. granulosus* s.s. (2)	2 (5)	2 (14)	190 (10)	10
–	–	–	0 (0)	11
*E. granulosus* s.s. (1)	1 (1)	1 (8)	65 (4)	12
–	–	–	31 (1)	13
–	–	–	61 (0)	14
*E. canadensis* G6/7 (10)	1 (10)	1 (13)	40 (1)	15
–	–	–	182 (4)	16
			119 (3)	17
Taeniidae eggs	Not amplified	2 (16)	352 (11)	18
–	–	–	210 (0)	19
–	–	–	16 (1)	20
*T. hydatigena* (1)	1 (1)	1 (1)	75 (3)	21
–	–	–	276 (10)	22
–	–	–	1039 (2)	23
–	–	–	238 (6)	24
–	–	–	3 (0)	25
	7 (30)	9 (78)	5607 (83)	Total

^a^*n* Number

**Table 2:** Different features of the representative sequences of four species of taeniids obtained in the present study

Accession No	Species	Gene	Query coverage (%)	Identity (%)	Ref
PQ035170	*E. granulosus s.s*	nad1	100	99.23	OQ355536.1
PQ035171	*E. canadensis G6/7*	nad1	100	98.57	MN340039.1
PQ060472	*T. serialis*	cox1	99	98.76	NC_021457.1
PQ060473	*T. hydatigena*	cox1	100	98.20	PP387596.1
PQ035169	*T. hydatigena*	nad1	98	97.10	MN175586.1

## Discussion

The purpose of the present study was to evaluate the risk of contamination with taeniid eggs caused by dogs in an urban setting in Kerman province. Four different species of taeniids were identified including *E. granulosus* s.s., *E. canadensis* G6/7, *Taenia hydatigena* and *T. serialis*. CE is mostly considered as a rural disease, the significance of the disease in urban environments is not investigated in many endemic countries. Some studies have shown that the parasite can circulate in urban areas. In Australia, dingoes were found infected in urban areas of Queensland, representing a potential public health hazard to the urban population [32]. In Chile, prevalence of *E. granulosus* s.l. was found higher in urban than in rural areas. Compared to the rural regions, urban settings have traditionally been considered as epidemiologically irrelevant areas for CE. Urban lifestyles are thought to be less conducive for maintaining the parasite's life cycle [20].

Our study highlights that transmission of *E. granulosus* s.l. can occur in certain urban areas where conditions are optimal for the maintenance of echinococcosis in dogs. These conditions include a high population of free roaming dogs, access to infected viscera through home slaughter in urban areas, lack of responsible dog ownership, environmental sanitation and lack of responsible feeding of FRDs [33]. It has been shown that free-roaming dogs are widely distributed in some urban and suburban areas of Kerman (Additional file 1: Fig. S1 a–b) [13]. There is widespread practice of home slaughter in different cultural and religious events as well as the presence of unofficial abattoirs in Iran. Sub-standard abattoirs also contribute to this problem [12]. The dogs roaming in the city and suburbs of Kerman live on the household waste dumps in the streets and passageways as well as on the foods provided by some local people. No slaughterhouse is active inside the city, however, there are several illegal abattoirs in the suburbs near the city with possible access for dogs to offal (Additional file 1: Fig. S1 c–d). It should also be noted that many households in the city practice home slaughter in some special cultural and religious occasions [34]. Lack of awareness of the risks associated with infected viscera increases the chance of transmission of CE.

Notably, in the present study the density of dog feces was higher in the residential areas of western and eastern parts of the city, where the human and dog population are higher than the central parts where most of the commercial and administrative buildings are located (Fig. 1b, Fig. 2). As the taeniid eggs were found in almost all sections of the city, the risk of CE to the people living in the city is potentially high, however as CE transmission is strongly associated with human behavior and lifestyle, as well as the viability of *E. granulosus* eggs in the environment [35]. Further in-depth studies are required to improve our understanding of CE epidemiology in urban regions including studies on the viability of taeniid eggs in the environment.

The authors observations and findings obtained from the National CE Registry (HydatidReg.ir) indicate that 54.5 of CE patients are living in urban regions of Kerman province [11]. It has been shown that the spread of *Echinococcus* eggs in urban areas is related to human activities like home slaughter and feeding offal to dogs [36]. Urbanization of alveolar echinococcosis (AE) has already been documented in Europe. In Zurich, foxes infiltrating into the city as well as increased preying of dogs on infected rodents presents particular risk of urban transmission of AE [37].

Little is known about the urban interactions of different species and genotypes within *E. granulosus* s.l. in the endemic regions. In a study in the suburbs nearby the abattoir of the city of Kerman in 2014, the prevalence of *E. granulosus* s.l. in dogs was estimated at 6.8% [16]. In another study on free-roaming dogs in Kerman and suburbs in 2013, 10.7% of FRDs necropsied were found infected and all *Echinococcus* isolates were identified as the G1 genotype of *E. granulosus* s.s. [17]. Among the neighboring countries, using molecular biological techniques in Turkey, the prevalence of *E. granulosus* ranged from 0.8 to 14% [38–41].

According to studies carried out in the past 15 years, the prevalence of *Echinococcus granulosus *sensu lato in free-roaming dogs in other parts of the country ranges between 0.36 and 38% [42, 43]. Unfortunately, very few studies have identified the parasites to the genotype level in the urban areas. In our study, *E. granulosus* s.s. and *E. canadensis* G6/7 genotypes were found perpetuating in the city. Interestingly both *E. granulosus* s.s. and *E. canadensis* G6/7 were found in FRDs of the same city block. Iran is a country with a diverse geography and climate. The existence of a diverse range of ruminant intermediate hosts in the country and the widespread livestock husbandry have led to the presence of several host-adapted genotypes in the region, among which the G1 genotypes of *E. granulosus* s.s. is the most frequent, followed by the G6 genotype of *E. canadensis* [44]. Similarly in many endemic regions of the world, G1 is the most common genotype identified in human and animals, particularly in sheep [45].

The second most frequent species in Iran, *E. canadensis* G6, is predominantly distributed in southeastern Iran, where camels are the main intermediate host [46]. Also in the Kerman province, *E. granulosus s.s.* G1 is the dominant genotype in sheep and goat, and *E. canadensis* G6 the main genotype found in camels, both occurring at relatively high frequency [47–49]. The fact that both species have been detected in dogs in the city indicates the overlapping sheep-dog and camel-dog cycle of these parasites in this region. This is in line with the findings of a study on human formalin-fixed paraffin-embedded samples in Kerman province, in which a high frequency of the both *E. canadensis* G6 (45.8%) and *E. granulosus* s.s. (54.2%) were recoded [50].

This study was focused on CE as one of the most important parasitic zoonoses in the people living in the Middle East and North Africa. We therefore used the sequential sieving method for detecting taeniid eggs including all kind of *Echinococcus* and *Taenia* eggs. It should be noted that this method excluded the possible detection of larger helminth eggs from the beginning. Therefore the data presented in this study do not reflect the true presence or absence of other helminth families infecting dogs. This issue should be considered in future works on free-roaming dogs. It should also be noted that PCR-sequencing for molecular identification of a single taeniid egg at the species level in the old dried feces is difficult, therefore we could only provide an accurate and reliable estimate of the prevalence of *E. granulosus* s.l. infection in fresh fecal samples.

Two other taeniid tapeworms were also detected in the present study, i.e. *Taenia hydatigena* (3.6%) and *T. serialis* (1.2%). *Taenia hydatigena*, whose larval stages cause cysticercosis in small ruminants, is a common dog tapeworm in Iran [51, 52]. Sheep and goats are frequently found infected with both *E. granulosus* s.l. and *T. hydatigena*. The presence of *T. hydatigena* in the urban dogs is additional evidence of a domestic sheep-dog cycle in an urban environment and confirms the continuous helminth transmission from livestock to dogs. The metacestode of *T. hydatigena* develops in the subserosa of the abdominal cavity primarily in the greater omentum but also on other sites such as the liver of sheep [53, 54]. Regarding the common practice of home slaughter in the province, that is not legal and is discouraged by health officials, free-roaming dogs have apparently regular access to the infected viscera of livestock in and around the city.

In our study, a dog sample with mixed infection of *Taenia serialis* and *T. hydatigena* was found in the western part of the city. *Taenia serialis* is a tapeworm transmitted between canids and lagomorphs including both rabbits and hares. In a worldwide scale, in the past 30 years, two cases of human infection with Coenurus serialis have been reported in intramuscular tissues [55]. In northeast of Iran two out of five rabbits were found infected with *T. serialis* metacestode, Coenurus serialis [56]. There is one report of *T. serialis* infection in dogs from the southwestern province of Khuzestan in Iran [4]. Free-roaming dogs have been documented as potential predators of hares and rabbits [57]. Our findings indicate that dogs can be involved in a synanthropic cycle of *T. serialis* in urban areas. Rabbits are kept as pet animals in many households and they are sold in the city pet shops. However, wildlife plays a more prominent role in *T. serialis* life cycle. Although human infections with *T. serialis* are rare, but regarding the zoonotic nature of *T. serialis* and development of the coenuri (metacestodes) in various human tissues, they still pose a minor but existing additional health risk to the people living in the city of Kerman, by acquiring infection through accidental ingestion of *T. serialis* eggs.

## Conclusions

This study documented the presence of *E. granulosus* s.s. eggs in dog feces collected in the city of Kerman. Certain urban conditions including lack of effective and humane dog population management, home slaughter in urban areas, feeding dogs with offal, lack of responsible dog ownership, regular deworming of owned dogs, and environmental sanitation may enhance the chance of transmission to the people living in the city. Further studies on *E. granulosus* s.l. in the free roaming dogs in urban areas are essential to improve our understanding of the epidemiology of CE in endemic countries. Findings of such studies provide background information to minimize the risk of CE transmission to humans and to control the disease in urban settings.

### Supplementary Information

Additional File1 Figure S1. Images demonstrating the situation facilitating the urban transmission of cystic echinococcosis in Kerman, Iran. a. Livestock husbandry on the outskirts of the city. b. Free-roaming dogs within the city. c and d. An unregistered abattoir near the city with free-roaming dogs waiting outside for food

## Data Availability

No datasets were generated or analysed during the current study.

## Abbreviations

FRDs: Free-roaming dogs
CE: Cystic echinococcosis
nad1: NADH dehydrogenase subunit
AE: Alveolar echinococcosis
