# Supplementary material for: Environmental contamination with feces of free-roaming dogs and the risk of transmission of Echinococcus and Taenia species in urban regions of southeastern Iran
Source: Parasit Vectors. 2024 Aug 23;17:359. doi: 10.1186/s13071-024-06435-x (PMC11342595; doi:10.1186/s13071-024-06435-x)
Supplement: Supplementary file 1 — Additional File1 Figure S1. Images demonstrating the situation facilitating the urban transmission of cystic echinococcosis in Kerman, Iran. a. Livestock husbandry on the outskirts of the city. b. Free-roaming dogs within the city. c and d. An unregistered abattoir near the city with free-roaming dogs waiting outside for food [file 13071_2024_6435_MOESM1_ESM.docx]

**Supplementary Information**

**
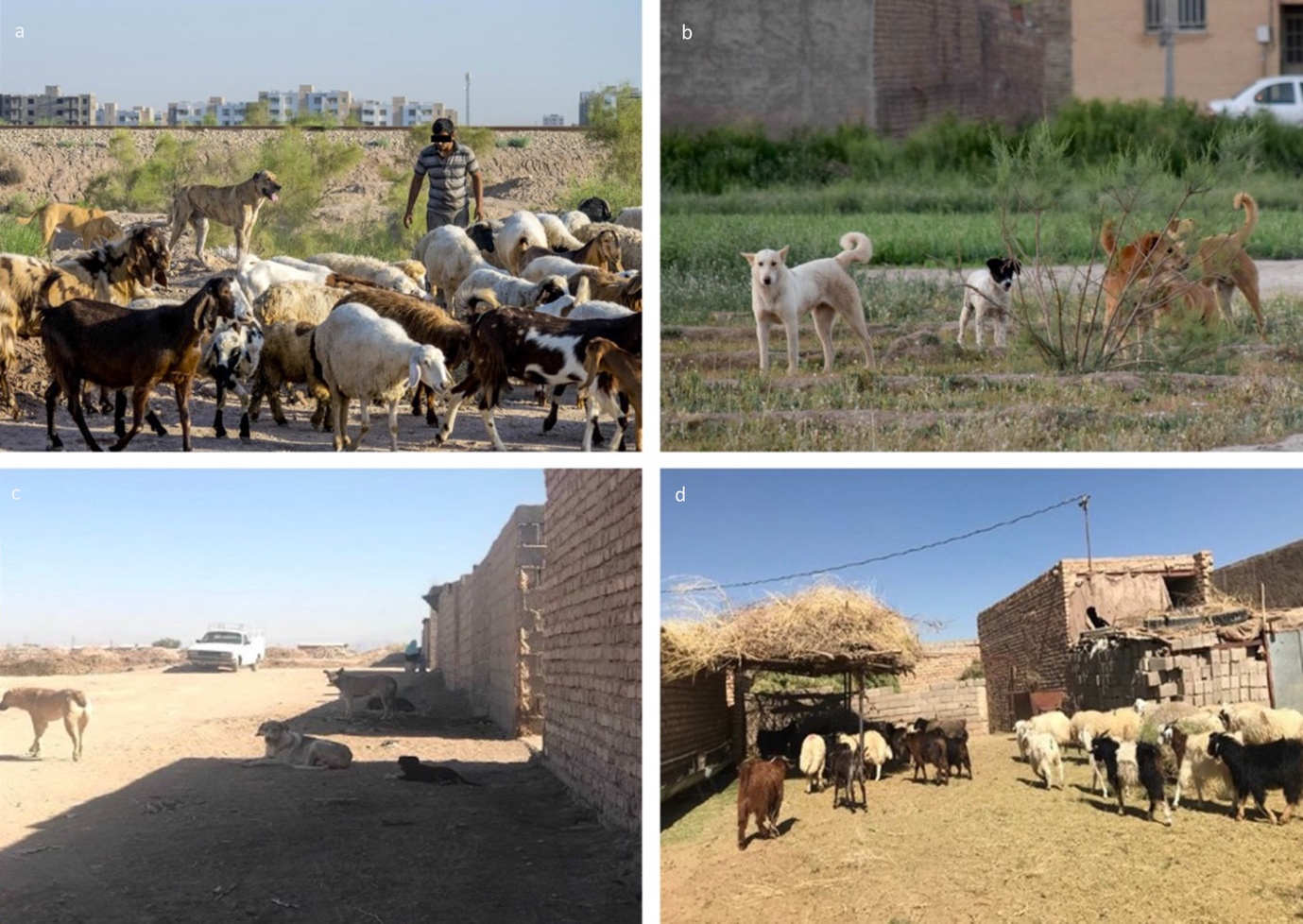
**

**Additional file 1: Figure S1.** Images demonstrating the situation facilitating the urban transmission of cystic echinococcosis in Kerman, Iran. a. Livestock husbandry on the outskirts of the city. b. Free-roaming dogs within the city. c and d. An unregistered abattoir near the city with free-roaming dogs waiting outside for food.
